# Transition bias influences the evolution of antibiotic resistance in *Mycobacterium tuberculosis*

**DOI:** 10.1371/journal.pbio.3000265

**Published:** 2019-05-13

**Authors:** Joshua L. Payne, Fabrizio Menardo, Andrej Trauner, Sonia Borrell, Sebastian M. Gygli, Chloe Loiseau, Sebastien Gagneux, Alex R. Hall

**Affiliations:** 1 Institute of Integrative Biology, ETH Zurich, Switzerland; 2 Swiss Institute of Bioinformatics, Lausanne, Switzerland; 3 Swiss Tropical and Public Health Institute, Basel, Switzerland; 4 University of Basel, Basel, Switzerland; Wageningen University, NETHERLANDS

## Abstract

Transition bias, an overabundance of transitions relative to transversions, has been widely reported among studies of the rates and spectra of spontaneous mutations. However, demonstrating the role of transition bias in adaptive evolution remains challenging. In particular, it is unclear whether such biases direct the evolution of bacterial pathogens adapting to treatment. We addressed this challenge by analyzing adaptive antibiotic-resistance mutations in the major human pathogen *Mycobacterium tuberculosis* (*MTB*). We found strong evidence for transition bias in two independently curated data sets comprising 152 and 208 antibiotic-resistance mutations. This was true at the level of mutational paths (distinct adaptive DNA sequence changes) and events (individual instances of the adaptive DNA sequence changes) and across different genes and gene promoters conferring resistance to a diversity of antibiotics. It was also true for mutations that do not code for amino acid changes (in gene promoters and the 16S ribosomal RNA gene *rrs*) and for mutations that are synonymous to each other and are therefore likely to have similar fitness effects, suggesting that transition bias can be caused by a bias in mutation supply. These results point to a central role for transition bias in determining which mutations drive adaptive antibiotic resistance evolution in a key pathogen.

## Abstract 2

Some types of mutations occur more frequently than expected. This study shows that such bias —an excess of transitions over transversions—influences the evolution of antibiotic resistance in a key global pathogen, Mycobacterium tuberculosis.

## Introduction

Mutation creates genetic variation and therefore influences evolution. Mutation is not an entirely random process but rather exhibits biases toward particular DNA sequence changes. For example, a bias toward transitions (purine-to-purine or pyrimidine-to-pyrimidine changes), relative to transversions (purine-to-pyrimidine or pyrimidine-to-purine changes), has been widely reported among experimental studies of the rates and spectra of spontaneous mutations, including those employing reporter constructs [1–4], and also studies of mutations spreading under relaxed selection, such as mutation accumulation experiments [5–13], comparisons of orthologous sequences at putatively neutral sites [14–17], and analyses of single-nucleotide polymorphisms within species [18]. Mutation biases also play an important role in models of neutral evolution [19,20], such as models of the evolution of intron density [21] and the genetic code [22].

Demonstrating the role of transition bias in adaptive evolution remains challenging, with most existing evidence derived from individual case studies [23–27]. Stoltzfus and McCandlish recently reported the first systematic study of transition bias in putatively adaptive evolution, using multiple criteria to identify adaptive mutations observed in experiments or nature, including their repeated occurrence in different lineages [28]. Their meta-analysis provides compeling evidence that transition bias influences adaptive evolution, with transitions observed in at least 2-fold excess of the null expectation that they occur once for every two transversions. Due in part to the difficulty of identifying definitively adaptive mutations, it remains unclear whether this bias applies in other adaptively evolving organisms. In particular, we do not yet know whether transition bias plays a role in populations of bacterial pathogens evolving in nature, such as those evolving resistance to antibiotics via chromosomal mutation. Identifying transition bias in such scenarios would improve both our basic understanding of how resistance evolves and our ability to predict the relative likelihoods of alternative mutational pathways to resistance. For example, if pathogen populations fix the first resistance mutation that appears (“first-come-first-served”) [29], then a mutation supply biased toward particular types of mutations will influence which genetic changes drive adaptation. Alternatively, if many beneficial mutations are available to selection and pathogens fix those with the highest selective advantage (“pick-the-winner”), a bias in mutation supply would have a weaker impact on which genetic changes drive adaptation.

An additional challenge in studying transition bias among adaptive mutations is determining whether an overabundance of transitions is due to a bias in mutation supply (i.e., mutation-based transition bias) or to a greater selective advantage conferred by transitions relative to transversions (i.e., selection-based transition bias). For example, mutation-based transition bias may be caused by the spontaneous deamination of cytosines to thymines [30], causing transitions to occur more frequently than transversions, whereas selection-based transition bias may be caused by differential fitness effects at either the nucleotide- or amino acid–level. At the nucleotide level, traits that are dependent upon DNA geometry, such as transcription factor binding [31], are less likely to be disrupted by transition mutations than transversion mutations [32]. The reason is that purines and pyrimidines differ in size, and transition mutations are therefore less likely to cause conformational changes to the DNA double helix [32]. At the amino acid level, nonsynonymous transitions may confer a greater selective advantage than nonsynonymous transversions because they are more likely to conserve the biochemical properties of amino acids [33]. Discriminating between mutation-based and selection-based transition bias has proven challenging to date [34,35].

Here, we study transition bias in the evolution of antibiotic resistance in *Mycobacterium tuberculosis* (*MTB*), a major human pathogen for which antibiotic resistance evolution via chromosomal mutation is a key obstacle to effective treatment [36]. Previous work suggests that *MTB* exhibits genome-wide mutation-based transition bias, with analyses of mutations spreading under relaxed selection [18,37] and during infection in cynomolgus macaques [38] and humans [39], reporting transition bias in at least 2-fold excess of the null expectation that one transition occurs for every two transversions. We aimed to determine whether such bias influences adaptive evolution in *MTB* and, in particular, the evolution of antibiotic resistance using two independently curated data sets of mutations (one of which we compiled for this study) that are known to confer antibiotic resistance and are therefore definitively adaptive. Additionally, we tested whether transition bias could be explained by transitions and transversions encoding amino acid changes with different average fitness effects by testing whether the observed bias was reduced among two subsets of adaptive mutations for which we can exclude this type of effect: 1) mutations located in gene promoters and in the 16S ribosomal RNA gene *rrs*, which is not translated to protein and therefore should not be influenced by selection-based bias caused by transitions encoding different amino acid changes than transversions, and 2) mutations that are synonymous to each other and are therefore likely to have similar fitness effects.

Our results reveal strong transition bias in the mutational paths to antibiotic resistance and in the number of times each mutational path is used in the evolution of antibiotic resistance across 22 genes or gene promoters that confer resistance to 11 antibiotics. We also observe transition bias among adaptive mutations that do not code for amino acid changes and among adaptive mutations that are synonymous to each other, consistent with the hypothesis that transition bias is at least partly mutation based. We therefore demonstrate that transition bias influences the evolution of antibiotic resistance in a key global pathogen.

## Results

### Antibiotic resistance mutations in *MTB*

We curated a data set of 152 unique point mutations that confer resistance to at least one of 11 different antibiotics and appeared in at least one of 9,351 publicly available *MTB* genomes (Materials and methods). We refer to this as the Basel data set. We also analyzed an independently curated data set of 208 unique point mutations that confer resistance to at least one of eight antibiotics and appeared in at least one of 5,310 *MTB* genomes [40] (Materials and methods). We refer to this as the Manson data set. The Basel and Manson data sets have 64 point mutations in common and together include resistance mutations for 11 antibiotics.

### Transition bias among mutations in both data sets

**Fig 1:**
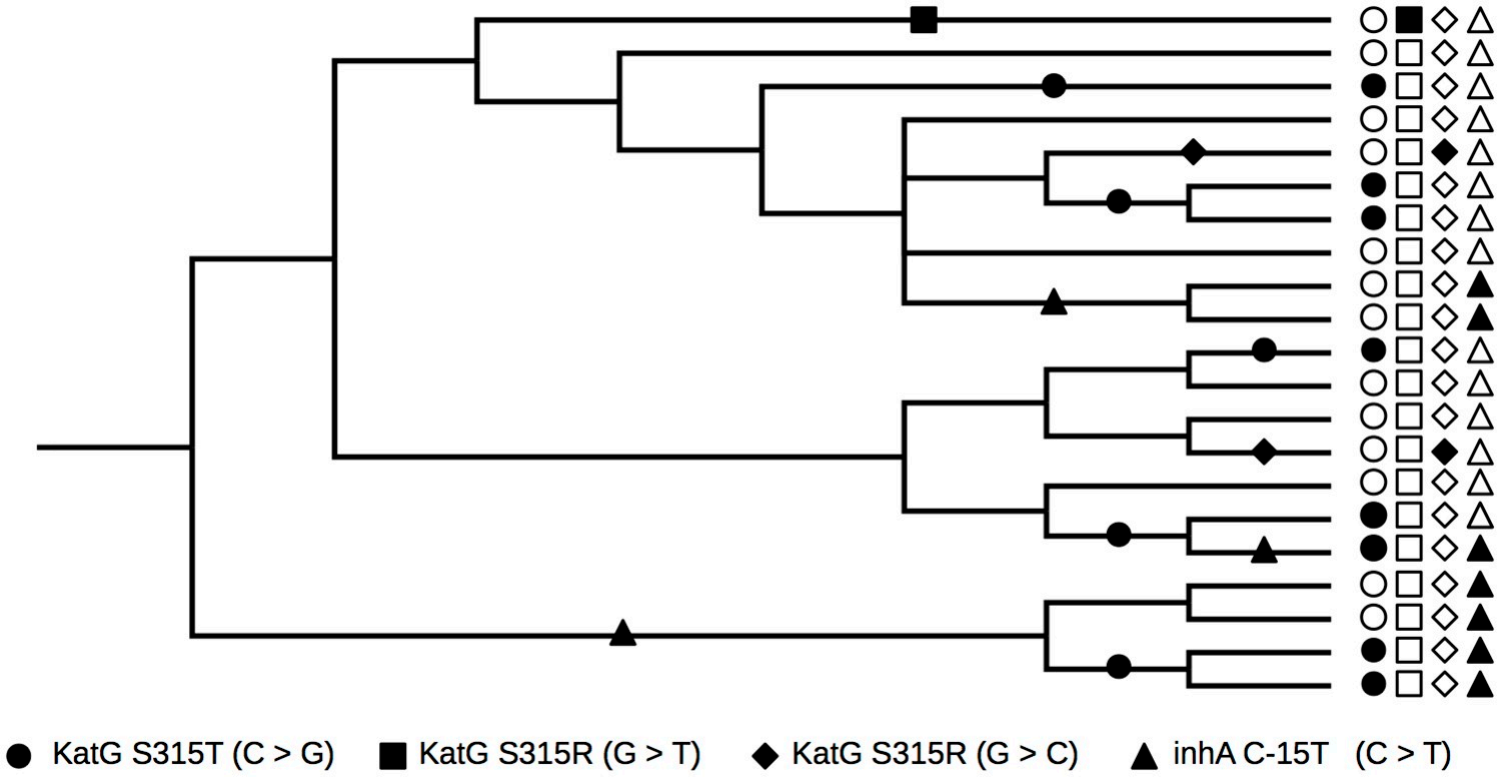
Schematic illustration of mutational paths and events. Four mutational paths that each confer resistance to isoniazid are shown as symbols (see legend at bottom) to the right of a hypothetical phylogenetic tree for 21 *MTB* strains. Full symbols represent derived genotypes, whereas empty symbols represent ancestral genotypes. The full symbols on the tree represent the reconstruction of the mutational history of the sample. The well-known S315T mutation in *katG*, encoded by a C > G transversion, is found in eight strains and, in this hypothetical reconstruction, has evolved independently five times. Thus, there are five events for this one mutational path. *MTB*, *M*. *tuberculosis*.

Following Stoltzfus and McCandlish [28], we separately studied transition bias in mutational paths and in mutational events (Fig 1). A mutational path is any single mutation in one of our data sets, such as the C > G transversion that causes the S315T amino acid change in the sole *MTB* catalase KatG to confer resistance to isoniazid [41]. We studied the Basel and Manson data sets separately, such that the 64 mutational paths that occur in both data sets were counted separately for each data set. Each mutational path may be used any number of times during adaptation of *MTB* strains to antibiotics, e.g., in different patients or in different geographic regions. Each independent occurrence is a mutational event. Multiple observations of the same mutational path in either of our data sets (the same mutation appearing on multiple *MTB* genomes) could result from independent mutational events, or from a single mutational event, the descendents of which are sampled multiple times. We accounted for this by calculating the number of mutational events (independent occurrences of each mutational path) for the Basel data set using a parsimony-based analysis of mutational gains and losses at all nodes in the reconstructed phylogeny of the 9,351 *MTB* genomes (Materials and methods; Fig 1). The Manson data set also contained estimates of the number of mutational events, derived using similar methods (Materials and methods). In total, the Basel and Manson data sets comprised 2,775 and 2,671 events, respectively.

We studied transition bias by calculating the transition:transversion ratio in each data set, separately for mutational paths and events. We calculated 95% binomial confidence intervals on this ratio and an empirical *P* value that describes the probability of observing a transition:transversion ratio greater than the observed ratio, given a null model (Materials and methods). As our default null model, we chose the most conservative among several alternative models described by Stoltzfus and McCandlish [28], which assumes that all nucleotide mutations are equally likely, and there is no difference between the average fitness effects of transitions and transversions. This null model gives a transition:transversion ratio of 0.5 because for any given nucleotide, there is one possible transition and two possible transversions. In some analyses at the level of mutational events, we adjusted the default null model, assuming instead a transition:transversion ratio equal to that observed at the level of mutational paths (accounting for the influence of path-level bias on event-level bias) and/or conserving the observed distribution of events per path (accounting for overrepresentation of individual paths).

**Fig 2:**
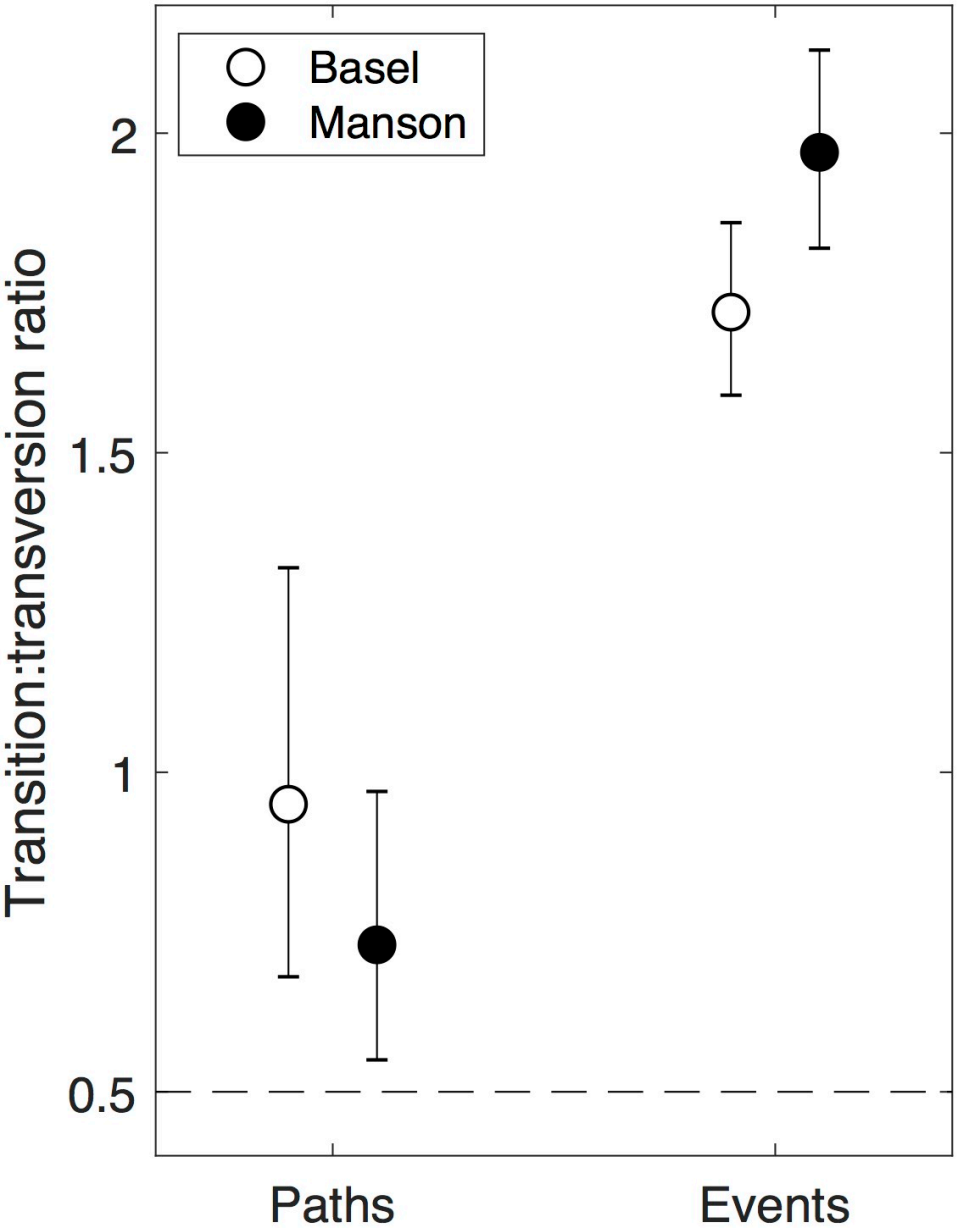
Transition bias in mutational paths and mutational events in the Basel and Manson data sets. Symbols represent transition:transverion ratios (Basel paths: 74:78, empirical *P* value = 7.1 × 10^−5^; Manson paths: 88:120, empirical *P* value = 4.2 × 10^−3^; Basel events: 1,755:1,020, empirical *P* value < 10^−6^; Manson events: 1,771:900, empirical *P* value < 10^−6^). Error bars represent 95% binomial confidence intervals. The dashed horizontal line shows the null expectation of the transition:transversion ratio, assuming our default null model that one transition occurs for every two transversions and that all mutations are independent. For additional null models used at the level of events, see the main text. The data visualized in this and all subsequent figures are presented in numerical form in S1 Data.

We observed transition bias among mutational paths and mutational events for both the Basel and Manson data sets (Fig 2). This bias was more pronounced among mutational events than mutational paths, in that the observed transition:transversion ratio at the event level was more than 3.4 times the null expectation that one transition occurs for every two transversions in both data sets (empirical *P* value < 10^−6^) and more than 1.4 times the null expectation at the path level (empirical *P* value < 0.004).

To determine whether the bias in mutational events goes beyond what we would expect given the observed bias in mutational paths, we performed three additional tests (Material and methods). First, we considered a revised null model that assumes the transition:transversion ratio among events equals the observed ratio among mutational paths (e.g., 0.95 in the Basel data set rather than 0.5 in the original null model). Under this null model, the probability of observing the transition:transversion ratios for mutational events was less than 10^−6^ for both the Basel and Manson data sets, indicating that the event-level bias is not explained by the path-level bias alone. In the second, more stringent test, we considered a null model that conserves the number of events observed for each path but randomly reassigns each path to be a transition or transversion with a probability determined by the transition:transversion ratio of 0.5 (Materials and methods). That is, this model assumes the observed variation in the number of events per path (which causes the stronger transition bias at the level of events compared to paths in our data sets) is independent of whether each path is a transition or a transversion. This is important because the observed transition bias among events could potentially be explained by a small number of paths that happen to be transitions recurring with high frequency (“jackpot mutations”), which could result from mutation-based biases, such as mutational hotspots, or from selection-based biases, such as mutations that confer higher resistance levels or have lower pleiotropic effects. Under this null model, the probability of observing the transition:transversion ratios for mutational events was 0.003 in the Basel data set and 0.001 in the Manson data set. Thus, jackpot mutations did not account for the increased event-level transition bias relative to the expectation that one transition occurs for every two transversions. In the third, most stringent test, we considered a null model that conserves the number of events observed for each path but randomly reassigns each path to be a transition or transversion with a probability determined by the transition:transversion ratio observed at the level of paths (Materials and methods). Under this null model, the probability of observing the transition:transversion ratios for mutational events is 0.09 in the Basel data set and 0.01 in the Manson data set. In summary, transition bias in mutational events tended to exceed what we would expect given the observed bias among paths, although in the Basel data set this excess was partly explained by a small number of jackpot mutations, with five mutational paths accounting for 52% of the events (S1 Fig). For example, the transition causing S450L in *rpoB* that confers resistance to rifampicin was represented by 272 events (16%), whereas the median number of transition events per mutational path was four (0.2%).

**Fig 3:**
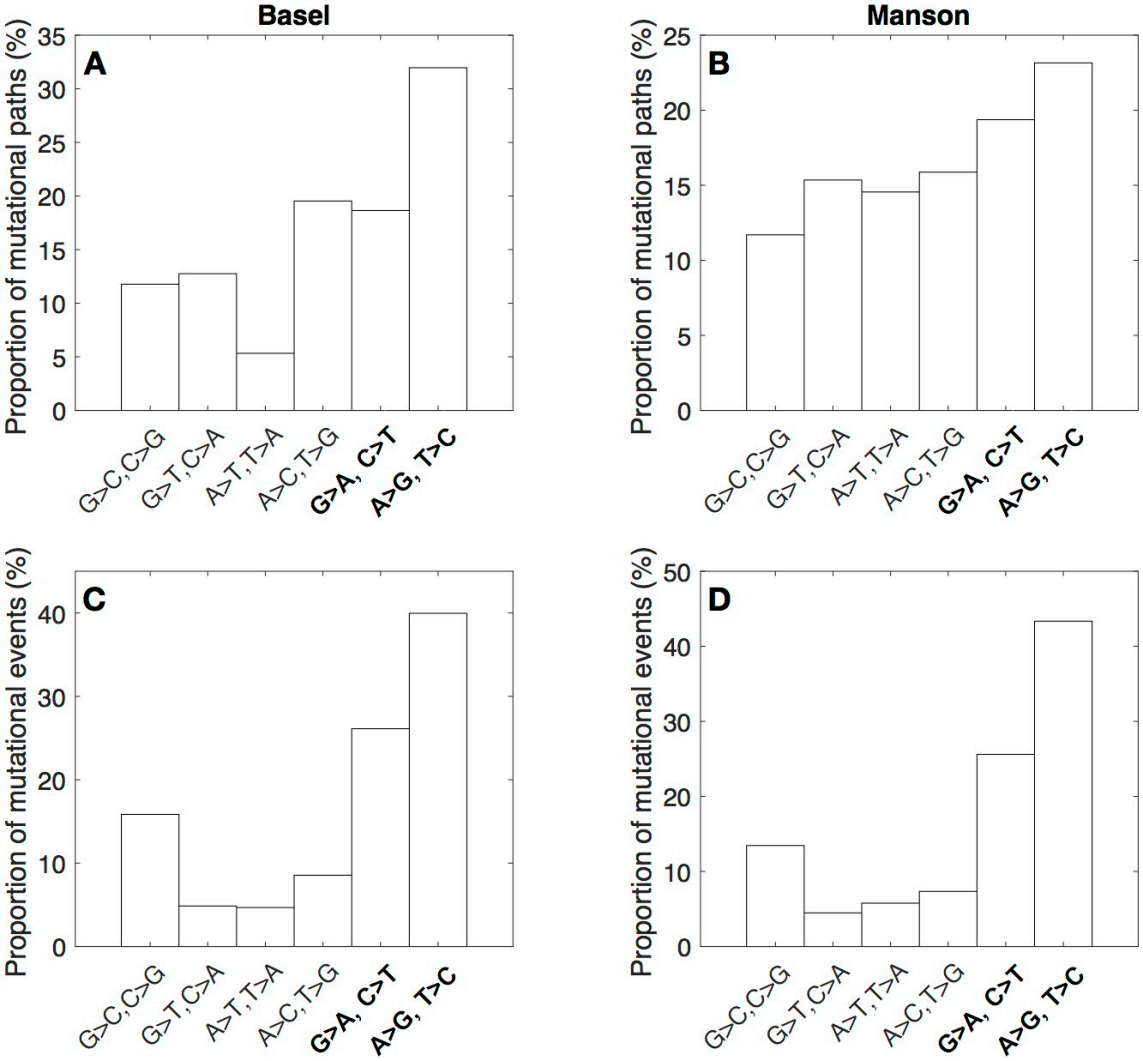
Relative rates of the six nucleotide pair mutations for mutational paths and events in the Basel and Manson data sets. Transitions are indicated with bold text. Rates adjusted for GC content (Materials and methods).

To determine which types of nucleotide changes explained the observed transition bias, we calculated the relative rates of all six possible nucleotide pair mutations, accounting for GC content [18], which is relatively high in *MTB* compared to other bacteria (Materials and methods). For mutational paths, the rate of A/T > G/C transitions exceeded the rate of any other nucleotide pair mutation (Fig 3A and 3B). The same was true of mutational events (Fig 3C and 3D), for which the rates of both forms of transitions (G/C > A/T and A/T > G/C) were at least 1.5 times that of all forms of transversions.

We next compared the relative rates of each type of mutation in our antibiotic-resistance data set to those observed for all mutations (not just resistance mutations) in the same 9,351 *MTB* genomes. These data comprise 325,714 mutational events along 305,316 paths, with a transition:transversion ratio of 1.91 (95% binomial confidence interval: [1.90, 1.93]; empirical *P* value < 10^−6^ for all null models). This is consistent with the event-level transition:transversion ratios observed in the Basel and Manson data sets. However, at the level of individual types of mutations, G/C > A/T transitions occurred more frequently than A/T > G/C transitions (accounting for GC content; S2A Fig), in contrast to our resistance data sets but consistent with a previous study of genome-wide mutations spreading under relaxed selection in several other bacteria, including *MTB* [18]. To better understand this potential discrepancy, we resampled the genome-wide mutational events 10^5^ times, each time with a sample size equivalent to that of the Basel data set and controlling for the distribution of events per path (S2B Fig; Materials and methods). We found the event-level relative rates of individual mutations in these resampled, genome-wide data sets were statistically indistinguishable from those in the Basel data set (S3 Fig). This indicates that the transition bias we observed among antibiotic-resistance mutations is consistent with the underlying genome-wide bias and that the different rates of individual nucleotide pair mutations can be explained by sample size and the distribution of events per mutational path. Note, we did not compare the Basel and genome-wide data sets directly at the level of mutational paths because the number of times each path is sampled (number of events per path) is very different in the two data sets (in the genome-wide data set, 96% of paths are sampled only once; in the Basel data set, we average many events per path; S2B Fig). This is expected to influence the observed path-level rates of different types of mutations [28]. Specifically, when most observed paths are represented by one event only (as in the genome-wide data set), the transition:transversion ratio of paths and events is very similar and may be influenced by mutation-based transition bias. When individual paths are represented by many events (as in the Basel data set), the transition:transversion ratio among events is expected to approach the adaptive mutation rate, and transitions will be overrepresented if there is mutation-based transition bias. However, because such samples are likely to capture a greater fraction of all possible paths (in this case, possible paths to antibiotic resistance), the transition:transversion ratio of observed paths will tend toward the ratio for all possible unique adaptive mutations, which we expect to approximate 0.5 even when there is mutation-based transition bias [28]. This is supported by rarefaction analysis of how the path-level relative rates change depending on the total number of mutational events sampled (S4 Fig). As we discuss below, this also helps to explain our observation of stronger transition bias at the level of events compared to paths.

### Transition bias varies among types of antibiotic resistance

Because the influence of transition bias might depend on the mechanism of antibiotic resistance, we next tested for transition bias separately for different antibiotics. This reduced the number of mutational paths and events that could be analyzed in each test, so we first determined the antibiotics for which we had sufficient statistical power to ensure that an observed lack of transition bias was not due to reduced sample size (Materials and methods). This analysis revealed that at the level of mutational paths, our data sets were too small to test for transition bias of the strength observed in the entire data set for any individual antibiotic (S5A and S5B Fig). This is because at least 44 (Basel) and 118 (Manson) mutational paths would be required to provide sufficient statistical power, and the maximum number of paths per antibiotic was 30 (for streptomycin) in the Basel data set and 51 (for rifampicin) in the Manson data set (S6A and S6B Fig). In contrast, we were able to test for transition bias in the mutational events associated with resistance to individual antibiotics because only 14 and 12 events were required to provide sufficient statistical power in the Basel and Manson data sets (S5C and S5D Fig). For this analysis, we considered mutations that simultaneously conferred resistance to multiple antibiotics in separate categories. For example, some mutations in the gene Rv1484 (*inhA*) and its promoter confer resistance to both isonazid and ethionamide (five and six mutations, respectively, in the Basel and Manson data sets). To avoid counting such mutations multiple times, we analyzed them separately and did not include them together with mutations that conferred resistance only to isonazid or ethionamide. All but two individual antibiotics and two categories of multiple antibiotics, all from the Basel data set, had more than one mutational path with enough events to test for transition bias (S6C and S6D Fig).

With one exception, we observed transition bias in the number of mutational events associated with the evolution of resistance to all individual antibiotics, indicated by transition:transversion ratios ranging from 1.59 for pyrazinamide to 41.6 for kanamycin (i.e., from more than 3-fold to more than 80-fold excess of the null expectation that a single transition occurs for every two transversions; Table 1). The exception was isoniazid, which was dominated by a single C > G transversion (S315T in *katG*; S1 Fig), representing 409 of the 472 events associated with isoniazid resistance in the Basel data set (or 671 events if we include mutations in *inhA* associated with resistance to both isoniazid and ethionamide) and 321 of the 389 events associated with isoniazid resistance in the Manson data set (or 545 if we include *inhA*). Such overrepresentation of individual paths (jackpot mutations) could potentially also explain the observed transition bias for resistance to the other antibiotics. To test this, we revised the null model, randomly reassigning each path to be a transition or a transversion, with a probability of one transition for every two transversions but conserving the observed number of events for each path [28] (Materials and methods). Most antibiotics still showed significant transition bias under this null model (rifampicin and isoniazid + ethionamide in both data sets and pyrazinamide in Manson and kanamicin in Basel did not; Table 1), indicating that the transition bias for individual antibiotics is not fully explained by jackpot mutations.

**Table 1:** Summary of transition bias in mutational events per antibiotic in the Basel and Manson data sets. Rows are ordered by decreasing number of events in the Basel data set. Dashes indicate antibiotics for which there are no mutational events in the respective data set. Mutations that confer resistance to multiple antibiotics are reported separately and were not counted among the events conferring resistance to individual antibiotics. *P* values indicating deviation from the default null model and three revised null models (1: the default null model, 2: accounting for path-level bias, 3: accounting for jackpot mutations, and 4: accounting for both path-level bias and jackpot mutations; see main text). Significance (*P* < 0.05) is indicated for each test by the number of the corresponding null model (e.g., 1, 2 indicates statistical significance for null models 1 and 2).

	Basel data set					Manson data set				
Antibiotic	T_*i*_	T_*v*_	T_*i*_:T_*v*_	95% CI	*P*	T_*i*_	T_*v*_	T_*i*_:T_*v*_	95% CI	*P*
RIF	365	223	1.64	(1.38, 1.94)	1, 2	494	209	2.36	(2.01, 2.80)	1, 2
EMB	390	154	2.53	(2.10, 3.07)	1, 2, 3, 4	342	131	2.61	(2.13, 3.22)	1, 2, 3, 4
INH	42	430	0.10	(0.07, 0.13)	ns	54	335	0.16	(0.12, 0.22)	ns
SM	285	71	4.01	(3.08, 5.28)	1, 2, 3	254	72	3.53	(2.71, 4.65)	1, 2, 3
INH, ETH	231	31	7.45	(5.11, 11.22)	1, 2	137	18	7.61	(4.64, 13.23)	1, 2
FQ	166	57	2.91	(2.14, 4.01)	1, 2, 3	-	-	-	-	-
PZA	67	28	2.39	(1.52, 3.86)	1, 2, 3	46	29	1.59	(0.98, 2.61)	1, 2
KAN	52	9	5.78	(2.82, 13.34)	1, 2	208	5	41.6	(17.54, 129.46)	1, 2, 3
ETH	7	10	0.70	(0.23, 2.04)	ns	16	10	1.60	(0.68, 3.95)	1, 3
OFX	-	-	-	-	-	220	91	2.42	(1.89, 3.12)	1, 2, 3, 4

**Abbreviations:** AK, amikacin; CAP, capreomycin; EMB, ethambutol; ETH, ethionamide; FQ, floroquinolones; INH, isoniazid; KAN, kanamycin; ns, not significant; OFX, ofloxacine; PZA, pyrazinamide; RIF, rifampicin; SM, streptomycin; T_*i*_, number of transitions; T_*i*_:T_*v*_, transition:transversion ratio; T_*v*_, number of transversions.

Next, we tested whether the observed bias among events for individual antibiotics also goes beyond what we would expect if we account for both jackpot mutations (by conserving the observed number of events per path as in the last analysis) and also for the observed transition:transversion ratio among paths for each antibiotic (by reassigning paths as transitions or transversions with a probability determined by the observed ratio at the path level, as above for events across the entire data set). Only ethambutol in both data sets and ofloxacin in the Manson data set showed significant deviation from this null model (Table 1). In summary, the strong transition bias for individual antibiotics was in some but not all cases explained by either path-level bias or by particular paths recurring with high frequency.

### Transition bias independent of changes in amino acid sequence

The above results suggest transition bias influences the evolution of antibiotic resistance in *MTB*. However, it remains unclear whether this bias is mutation based or selection based. To disentangle these potential sources of transition bias, we used two different approaches. First, if the observed bias were caused by transitions and transversions encoding amino acid changes with different average fitness effects, we would not expect the bias to extend to mutations that do not encode amino acid changes. We tested this by examining mutations in gene promoters and the 16S ribosomal RNA gene *rrs*. There were 23 such mutations in the Basel data set and 25 in the Manson data set (12 occur in both data sets). Because this did not provide enough statistical power to perform the analysis at the level of paths, we only considered events here. We found an excess of transitions. Specifically, in the Basel data set, there were 603 events comprising 525 transitions and 78 transversions (transition:transversion ratio 6.73; 95% binomial confidence interval: [5.30, 8.65]; empirical *P* value < 10^−6^ for our default null model). In the Manson data set, there were 520 events that comprised 421 transitions and 99 transversions (transition:transversion ratio of 4.25; 95% binomial confidence interval: [3.41, 5.35]; empirical *P* value < 10^−6^ for our default null model). In both data sets, this transition bias was not explained by jackpot mutations (tested using a revised null model assuming a transition or transversion ratio of 0.5 but conserving the distribution of events per path: *P* = 0.0007 for Basel and *P* = 0.008 for Manson). However, in both cases, we found weaker evidence of transition bias when we also accounted for the observed transition:transversion bias among paths (tested using a revised null model assuming the observed path-level transition:transversion ratio and conserving the distribution of events per path: *P* = 0.18 for Basel and *P* = 0.03 for Manson). Thus, we found transition bias among events that do not encode amino acid changes, and this was driven at least partly by bias among the corresponding mutational paths. Note that mutations in promoters or genes such as *rrs* may nevertheless have variable fitness effects despite not encoding amino acid changes [32,42], and we discuss this further below.

Second, we considered cases in which different mutations caused the same resistance-conferring amino acid change and were therefore synonymous to each other and expected to have similar fitness effects. Specifically, we considered amino acid changes that can be caused by both transition and transversion mutations at the same ancestral codon. For example, methionine can mutate to isoleucine via the transition ATG > ATA or the transversions ATG > ATT and ATG > ATC. In the standard genetic code, there are five such amino acid changes (Table 2). These were rare or nonexistent in the Basel and Manson data sets (Table 2), except the amino acid change methionine-to-isoleucine, which occurred in three mutational paths and 137 events in the Basel data set and in four mutational paths and 135 events in the Manson data set. In the Basel data set, the 137 events comprised 88 transitions and 49 transversions (transition:transversion ratio of 1.80; 95% binomial confidence interval: [1.25, 2.60]; empirical *P* value < 10^−6^ for the default null model). In the Manson data set, the 135 events comprised 96 transitions and 39 transversions (transition:transversion ratio of 2.46; 95% binomial confidence interval: [1.68, 3.67]; empirical *P* value < 10^−6^ for the default null model). This shows transitions were also overrepresented among events conferring resistance via the same amino acid change (from methionine to isoleucine). These observations weigh against the selection-based hypothesis that transition bias is driven by different average fitness effects of amino acid changes caused by transitions and transversions. If this hypothesis were true, we would expect the transition:transversion ratios to decrease when we removed possible differences in the fitness effects of resulting amino acid changes. Instead, the transition:transversion ratios were similar to those for all events, indicating that mutation-based transition bias is sufficient to produce transition bias of the same strength as that observed in the Basel and Manson data sets. Note that for these mutations, the observed transition:transversion ratio at the path level is the same as in the default null model (0.5), and we could not account for jackpot mutations due to the small number of paths.

**Table 2:** Observed transitions and transversions in mutational events in the Basel and Manson data sets and in mutational paths in the TBDReaMDB data set among amino acid changes that can be caused by both transition and transversion mutations to the same codon.

Amino acid change	Ancestral codon	Possible Ti:Tv	Basel Ti:Tv	Manson Ti:Tv	TBDReamDB Ti:Tv
G → R	GGA	1:1	0:0	0:0	0:1
G → R	GGG	1:1	0:0	0:0	1:0
M → I	ATG	1:2	88:49	96:39	6:5
F → L	TTT	1:2	0:0	0:0	1:0
F → L	TTC	1:2	0:0	0:0	1:6
W → R	TGG	1:1	3:0	0:0	7:0
Stop → R	TGA	1:1	0:0	0:0	0:0

**Abbreviations:** T_*i*_, number of transitions; T_*i*_:T_*v*_, transition:transversion ratio; T_*v*_, number of transversions.

Ideally, we would have a sufficient number of mutational paths or events to study transition bias among mutations that are synonymous to each other for all of the amino acid changes in Table 2. To this end, we analyzed an older data set, TBDReaMDB [43], that included a greater number of resistance mutations but that was not compiled with the same strict inclusion criteria as the Basel and Manson data sets. Across the 717 mutational paths from this data set that we included (Materials and methods), we observed transition bias (transition:transversion ratio of 0.86; 95% binomial confidence interval: [0.74, 1.00]; empirical *P* value < 10^−6^ for the default null model). In this data set, there was also a sufficient number of the amino acid changes shown in Table 2 to take an aggregated approach to calculating transition bias among mutations that are synonymous to each other. Specifically, among all such mutational paths, there were 16 transitions and 12 transversions (Table 2; transition:transversion ratio of 1.33; empirical *P* value = 0.02). This was nearly twice the expected ratio of 0.67, derived from a null model accounting for the number of transitions and transversions in these particular mutational paths (Materials and methods).

## Discussion

We found strong transition bias at multiple levels (mutational paths and events) and in multiple independently curated data sets (Basel, Manson, and TBDReamDB) for resistance mutations that confer a fitness benefit in the presence of antibiotics. By focusing part of our analysis on mutations in gene promoters and the 16S ribosomal RNA gene *rrs* and on mutations that are synonymous to each other, we overcame the difficulty of ruling out an overabundance of transitions caused by transitions and transversions encoding amino acid changes with different average fitness effects. Our data also revealed notable exceptions to the general trend toward transition bias. In particular, the most common resistance mutation by far against isoniazid was a transversion. Our results have four key implications for antibiotic resistance evolution and mutational biases.

First, quantifying the overabundance of transitions improves our ability to predict mutational pathways to resistance. *MTB* often acquires multiple resistance mutations sequentially, and some mutational trajectories are more common than others [44]. Our results suggest the probability of following a given trajectory will be higher when it contains a greater fraction of transitions than alternative trajectories encoding similar resistance phenotypes.

Second, for at least part of our data, we excluded an important potential explanation for transition bias, specifically that transitions encode amino acid changes with different average fitness effects than transversions. We did this by showing that the bias extended to mutations in gene promoters and the 16S ribosomal RNA gene *rrs* and to mutations that are synonymous to each other. The transition bias for these mutational events was similar or even stronger compared to that for mutational events in the entire Basel and Manson data sets, suggesting that it is not necessary to invoke selection-based transition bias to explain the transition bias observed in these data sets. However, we cannot rule out variable fitness effects that are not linked to amino acid changes, such as those observed for streptomycin-resistance mutations in ribosomal genes [42] and synonymous resistance mutations in other bacterial species [45]. If the level of selection-based transition bias for these mutations is similar to that for mutations that cause amino acid changes, then this could also explain the similar transition bias observed for these two classes of mutations. However, earlier analyses of the effects of noncoding mutations on gene expression in reporter assays [32] and of the effects of missense mutations on fitness in viruses and bacteria [35] found at most a marginal difference in the effects caused by transitions relative to transversions. Therefore, while we do not argue there is no role for biased fitness effects in general (in fact there is evidence of this for viruses [34]), it is unlikely to be the sole cause of the overabundance of transitions we observed, indicating that this is explained instead or in addition by a higher mutation supply of transitions than transversions.

Third, if we accept that a biased mutation supply explains at least some of the observed bias among resistance-conferring mutational paths and events, this is consistent with a role for mutation-limited “first-come-first-served” dynamics [20,29] in resistance evolution in *MTB* [36]. This is also consistent with the strict clonality, small within-host effective population size upon infection and low mutation rate of *MTB*, such that infections expand slowly from a small infectious dose that initially contains little genetic variation [36,46]. However, genomic evidence shows that multiple resistant *MTB* genotypes can occur within the same patient [39,47], which is less supportive of mutation-limited dynamics. The extent to which such genotypes compete with each other depends on the spatial population structure, and if they are in different lung sections, they may not compete directly [48]. Overall, this indicates resistance may evolve via a process somewhere along a continuum between first-come-first-served and pick-the-winner [20].

Fourth, *MTB* may be closer to one end of this continuum when challenged with some antibiotics than others. Specifically, a single transversion accounted for >70% of the mutational events conferring isoniazid resistance, indicating a greatly reduced role for transition bias. Instead, the high frequency of the S315T mutation in *katG* among isoniazid-resistant isolates may reflect that it confers resistance to clinically relevant concentrations, particularly in combination with *inhA* promoter mutations [49], without severely reducing catalytic function [41]. *MTB* strains carrying this mutation, while not detected in in vitro mutant screens [50], also appear more likely to transmit than strains with other isoniazid-resistance mutations [51,52]. Therefore, this may represent a scenario in which the influence of mutation-based transition bias is greatly reduced because of strong selection for an individual transversion. To directly disentangle the contributions of mutational frequencies and fitness effects for isoniazid resistance would require experimental estimates of these parameters. However, interpreting such data would be challenging because we know the most important isoniazid resistance mutations do not spread in vitro as they do in vivo [50], including the *katG* S315T mutation that was so prevalent here. Such discrepancies may reflect general in vivo versus in vitro differences, like the increased importance of oxidative damage in the lung environment for mutation during infection [38]. Thus, although in vitro data captures some key aspects of resistance evolution in nature [53,54], a full understanding requires combining this with epidemiological data [55] or genomic analysis of natural and clinical strains, as we have done here.

Our observation that transition bias is prevalent at the level of paths but even stronger at the level of events is consistent with Stoltzfus and McCandlish’s (2017) observations for mutations in parallel experimental or natural populations and is expected under mutation-based transition bias when the sample size is large [28]. This is because in large samples, for which each path is represented by many events, the event-level ratio will tend toward that of the adaptive mutation rate, but the path-level ratio will tend toward the ratio among all possible unique adaptive mutations, which would approximate 0.5 even when there is mutation-based transition bias [28]. The observed ratio at the level of paths in our data sets, while weaker than at the level of events, still exceeded 0.5. This could be because our data sets are incomplete samples of the possible paths to resistance in *MTB*, and the likelihood of a given path featuring in our data sets is higher if it occurs more frequently (i.e., mutation-based bias and intermediate sample size). Alternatively, our data sets may capture the vast majority of paths to resistance, but due to selection-based bias, a greater fraction of them are transitions than transversions. The former possibility is consistent with the lower path-level transition:transversion ratio in the Manson data set, which has more paths than the Basel data set (S4 Fig). Additionally, we are probably closest to knowing all possible mutational paths to resistance for rifampicin, which had the most observed events in our data sets (S6 Fig) and has been used in fluctuation analyses for resistant mutants [37]. For this antibiotic, the path-level transition:transversion ratio approached 0.5 in the Basel and Manson data sets and in fluctuation analysis data for the most permissive antibiotic concentration in a previous study [37] and was higher at the event level in all cases (S1 Table).

Mutation bias comes in many forms [56]. In bacteria, these include deletion bias [57], increased mutation rates in specific sequence contexts [58], in genes that are highly transcribed [2], on the lagging strand [59], or farther from the origin of replication [60]. Direct studies of the mutation spectrum in *MTB* are limited, so it is not currently possible to ascertain whether these biases exist in *MTB* or how they might interact with transition bias to influence adaptive evolution. We therefore cannot rule out the possibility that such interactions explain some of the heterogeneity we observe in the number of mutational events per path. We note, however, that the six jackpot mutations of the Basel data set show no bias toward or away from the origin of replication (the maximum possible distance from the origin of replication is 2.2 Mb, and the distance of the mutations range from 0.16 Mb to 2.2 Mb); all are found in distinct sequence contexts (nucleotide triplets), four are found on the leading strand, and the other two are found on the lagging strand. Transcription level is also unlikely to explain the heterogeneity in the number of events per path, because each jackpot mutation occurs in a gene that has alternative resistance-conferring mutational paths, and these tend to have far fewer events. For example, *rrs* is among the most highly expressed genes in *MTB*. Its one jackpot mutation has 145 events, whereas the other seven resistance-conferring mutational paths in this gene have between one and 42 events (S4 Data).

Our finding that A/T > G/C transitions were the most common mutations in the Basel and Manson data sets appears to contrast with earlier evidence that G/C > A/T transitions are the most common type of mutation spreading under relaxed selection in multiple bacterial species, including *MTB* [18]. Further evidence of a distinctive transition bias toward G/C in mycobacteria, and in particular an overabundance of A/T > G/C transitions, comes from a recent mutation accumulation experiment with the related species *Mycobacterium smegmatis* [7]. Another earlier study testing for evidence that oxidative damage caused by antibiotics influences mutational biases also found that A/T > G/C transitions were the most common type of mutation in an earlier version of the TBDReaMDB [61]. However, by resampling mutations observed in the MTB genomes we analyzed, irrespective of whether they conferred antibiotic resistance, we found this discrepancy could be explained by the sample size of resistance mutations compared to all mutations and the distribution of events per path in the Basel data set. Thus, transition bias is pervasive across earlier studies and our data set.

In conclusion, our data support the hypothesis that a bias toward transitions plays a key role in determining the genetic changes driving antibiotic resistance evolution in *MTB*.

## Materials and methods

### The Basel data set

We curated a list of mutations known to confer resistance to one or more of the following drugs or drug classes: isoniazid, ethionamide, rifampicin, ethambutol, pyrazinamide, fluoroquinolones, and aminoglycosides. Starting from a previously published set of 120 mutations [62], we first excluded mutations in *rpsA* and *ahpC* because these genes were unlikely to confer resistance to pyrazinamide and isoniazid, respectively [63,64]. We then added *gyrB* to the list of pertinent genes because some mutations in this gene have been shown to lead to fluoroquinolone resistance [65].

We included additional mutations if they met one or more of the following criteria: 1) they have been shown, by virtue of allelic exchange, to confer resistance; 2) introduction of the mutation into the enzyme of interest was investigated in vitro and shown to confer properties consistent with drug resistance; 3) they were identified in laboratory-generated antibiotic-resistant strains as the most likely candidate for resistance; or 4) there was a clear correlation between the presence of the mutation and drug resistance as detected by phenotypic drug susceptibility testing of clinical strains. This resulted in a list of 196 mutations (S2 Data), of which we found 152 in at least one of 9,351 publicly available *MTB* genomes (S3 Data), which we obtained from Menardo and colleagues (2018) [66], including only genomes belonging to *MTB* sensu stricto. These are the mutational paths in the Basel data set (S4 Data). The transition:transversion ratio for the 196 mutations is 0.90 (95% binomial confidence interval: [0.68, 1.21]; empirical *P* value = 0.000023 for our default null model), which is similar to the transition:transversion ratio for the 152 mutations that were also found in at least one of the *MTB* strains (transition:transversion ratio = 0.95; 95% binomial confidence interval: [0.68, 1.32]; empirical *P* value = 0.000071 for our default null model).

We determined the mutational events by first calling single-nucleotide polymorphisms in the 9,351 genomes and then using the polymorphisms to reconstruct the genomes’ phylogeny and to infer mutational gains and losses throughout the phylogeny, as follows. We clipped Illumina adaptors, trimmed low-quality reads using Trimmomatic v. 0.33 (SLIDINGWINDOW:5:20) [67], and removed reads shorter than 20 base pairs. We merged overlapping paired-end reads using SeqPrep v. 1.2 (overlap size = 15) and mapped the resulting reads to the reconstructed ancestral sequence of the *MTB* complex [68] using the mem algorithm of BWA v 0.7.13 [69]. We marked duplicated reads using the MarkDuplicates module of Picard v. 2.9.1, performed local realignment of reads around indels using the RealignerTargetCreator and IndelRealigner modules of GATK v. 3.4.0 [70], and excluded reads with an alignment score lower than (0.93 × read_length)–(read_length × 4 × 0.07), which corresponds to more than seven mismatches per 100 base pairs. We called single-nucleotide polymorphisms using Samtools v. 1.2 mpileup [71] and VarScan v. 2.4.1 [72], with the following thresholds: minimum mapping quality of 20, minimum base quality at a position of 20, minimum read depth at a position of 7, minimum percentage of reads supporting the call 90%, and maximum strand bias for a position 90%. Genomes were excluded if 1) they had an average coverage <20x, 2) more than 50% of their single-nucleotide polymorphisms were excluded due to the strand bias filter, 3) more than 50% of their single-nucleotide polymorphisms had a percentage of reads supporting the call between 10% and 90%, or 4) they contained single-nucleotide polymorphisms that belong to different *MTB* lineages because this indicates that a mix of genomes was sequenced. Finally, we excluded all genomic positions with more than 10% missing data. This resulted in a final data set of 300,583 polymorphic positions.

We inferred a phylogenetic tree based on the 300,583 single-nucleotide polymorphisms with FastTree [73], using double digit precision and the options -nocat and -nosupport. We extracted the bases at each of the 152 genomic positions in our list of resistance mutations from the vcf file for the 9,351 genomes and assembled them in a multiple-sequence alignment. To determine the number of mutational events per mutational path, we reconstructed the nucleotide changes at the 152 genomic positions on the phylogenetic tree rooted with the inferred ancestral sequence of the *MTB* complex [68]. To do this, we used two maximum parsimony algorithms (ACCTRAN and DELTRAN) [74] implemented in PAUP* v. 4.0a [74], giving equal weight to all characters and considering them as unordered; we considered for further analyses only the events reconstructed by both algorithms.

### The Manson data set

Manson and colleagues [40] compiled a list of polymorphisms associated with resistance to eight antibiotics (S4 Table in [40]) and searched for these polymorphisms in 5,310 *MTB* genomes. They found 392 of these polymorphisms in at least one genome (S5 Table in [40]). We filtered these polymorphisms to only include point mutations, resulting in a data set of 208 mutational paths. Manson and colleagues [40] calculated the number of events per mutational path by reconstructing the phylogeny of the 5,310 *MTB* genomes and using a parsimony-based analysis to determine mutational gains and losses throughout the phylogeny. We used their estimates of the number of events per mutational path, as they were reported in S5 Table of [40].

### The TBDReaMDB data set

TBDReaMDB is a data set of 1,178 mutational paths associated with resistance to at least one of nine antibiotics. We filtered this data set to only include mutational paths that (1) are nonsynonymous point mutations, with both the ancestral and derived codons reported (709 paths); or (2) point mutations in promoters that are upstream of a gene’s transcription start site (eight paths); and (3) are nonredundant, where we considered two mutational paths redundant if they were the same mutational path and associated with the same gene ID, drug, and codon position (or nucleotide position in the case of promoters). The filtered data set contains 717 mutational paths.

We used this data set, which includes a greater number of mutations than the Basel and Manson data sets but with less strict inclusion criteria, to study transition bias among amino acid changes that can be caused by mutational paths that are transitions or transversions and that arise from the same codon (Table 2). For this purpose, we developed a null model accounting for the number of transitions and transversions in the available mutational paths that cause such amino acid changes. Specifically, for a given combination *i* of amino acid change and ancestral codon, there are *n*_*i*_ mutational paths that are transitions and *p*_*i*_ mutational paths that are transversions. In the TBDReaMDB data set, there are *m* = 6 such combinations of amino acid changes and ancestral codons (top six rows of Table 2). The expected probability of a transition under the null model is $\sum_{i\;=\;1}^{m}n_{i}/\sum_{i\;=\;1}^{m}\left(n_{i}+p_{i}\right)$. For the *m* = 6 observed combinations of amino acid change and ancestral codon, this gives a null transition probability of 6/15 = 0.4, which equates to a transition:transversion ratio of 0.67.

### Calculating confidence intervals for transition:transversion ratios

We calculated 95% binomial confidence intervals on the transition:transversion ratios of mutational paths and events using the Matlab function binofit.m, which takes as input the number of trials (i.e., the number of mutational paths or events) and the number of successes (i.e., the number of the paths or events that are transitions). It provides as output an estimate of the 95% confidence interval of the probability of success for the binomial distribution. The probability of success is equivalent to the probability a mutational path or event is a transition. We multiplied this probability for both the lower and upper bounds of the interval by the total number of mutational paths or events to determine the lower and upper bounds on the number of transitions. We then performed the analogous calculation for transversions and used these numbers to determine the lower and upper bounds of the 95% confidence interval for the transition:transversion ratio.

### Calculating empirical *P* values for transition:transversion ratios

To calculate an empirical *P* value for an observed transition:transversion ratio in a data set containing *x* mutational paths or events, we randomly generated 10^6^ data sets of *x* mutations according to our default null model that transversions are twice as likely as transitions. Each of the *x* mutations in each data set was chosen to be a transition with probability 1/3 or a transversion with probability 2/3 (when we analyzed mutations that are synonymous to each other in the TBDReaMDB data set, we used probabilities of 0.4 and 0.6, and in revised null models for some of the event-level analyses, we used probabilities equivalent to the observed path-level transition:transversion ratio, described further below). We determined the transition:transversion ratio for each of these data sets and then calculated the empirical *P* value as the fraction of these ratios that were greater than the observed ratio.

To disentangle different sources of transition bias among mutational events, we considered three revised null models. First, to determine whether event-level bias exceeded path-level bias, we used the same default null model as above but assigned mutations as transitions or transversions with a probability equivalent to the observed path-level transition:transversion ratio (0.49 or 0.42 for all paths in the Basel and Manson data sets), instead of one transition for every two transversions. Second, we controlled for “jackpot mutations” by conserving the distribution of mutational events per mutational path but randomly reassigning mutational paths as either transitions or transversions according to a transition:transversion ratio of 0.5 [28]. We repeated this process 10^6^ times as above to create a null distribution of the number of transition events, which we used to calculate an empirical *P* value. Third, we accounted for both jackpot mutations and path-level bias, using a null model that conserved both the distribution of events per path and the observed path-level transition:transversion ratio but randomly permuted the assignment of mutational paths as transitions or transversions (thus maintaining the transition bias at the level of paths), again repeating the process 10^6^ times to calculate an empirical *P* value. The latter two tests are most likely to reject the null hypothesis when transition events are uniformly distributed among the corresponding mutational paths. They are least likely to reject the null hypothesis when all transition events correspond to a single mutational path (i.e., a single jackpot mutation).

### Rarefaction analysis

We performed a rarefaction analysis to show how path-level transition bias depends upon the number of sampled events. For both the Basel and Manson data sets separately, we randomly sampled *x* mutational events and measured the transition bias in the resulting mutational paths. We repeated this process 10^5^ times for each of 50 logarithmically spaced values of *x* between 1 and the total number of events in the Basel and Manson data sets (2,755 and 2,671, respectively). The results of this analysis are shown in S4 Fig.

### Calculating the relative rates of the six nucleotide pair mutations

GC content may influence the number of mutational paths or mutational events in our data sets. *MTB* has a high GC content: 65.6% genome-wide [75] and 64.4% in the 17 genes associated with resistance in the Basel and Manson data sets. Thus, we expected to see more mutations from G/C or C/G than from A/T or T/A, simply because there are more Gs and Cs in the genes associated with resistance in our data sets. To control for this effect in our calculation of the relative rates of the six possible nucleotide pair mutations, we followed the method of Hershberg and Petrov [18]. Specifically, we first determined the number of mutations of each type we would expect under equal GC content by multiplying the number of mutations from A/T to G/C, C/G, or T/A by 64.4/(100 − 64.4) (or by 65.6/[100 − 65.6] for the genome-wide mutations). We then calculated the relative rates of the six nucleotide pair mutations by dividing the number of each (unchanged for mutations from G/C) by the sum of all possible pairs of mutations and multiplying by 100.

### Comparing the event-level relative rates of the six nucleotide pair mutations in the Basel data set to those observed genome-wide

In the Basel data set, the rate of G/C > A/T transitions was lower than the rate of A/T > G/C transitions, whereas the opposite was true genome-wide (S2A Fig). We sought to determine if this discrepancy could be explained by differences in sample size and in the distributions of events per path (S2B Fig). To do so, we took a sampling approach. We randomly chose 152 mutational paths from the 305,316 paths of the genome-wide data set. We then randomly assigned the number of events per path in the sample according to the distribution of events-per-path in the Basel data set. We then calculated the event-level relative rates of the six nucleotide pair mutations and repeated this process 10^5^ times, yielding a distribution of the relative rates (S3 Fig). For each relative rate, we calculated an empirical *P* value by determining the fraction of random samples that had a relative rate that was greater than (or less than, depending on the test) the value observed in the Basel data set. For example, for the relative rate of G/C > A/T, we determined the fraction of the 10^5^ samples that had a relative rate that was less than that observed in the Basel data set because in S2A Fig the height of the corresponding white bar is lower than the height of the corresponding black bar. In contrast, for the relative rate A/T > G/C, we determined the fraction of the 10^5^ samples that had a relative rate that was greater than that observed in the Basel data set because in S2A Fig the height of the corresponding white bar is higher than the height of the corresponding black bar.

### Calculating statistical power

The numbers of paths and events are much smaller for individual antibiotics than in the full data sets, which may render our test of transition bias statistically underpowered for individual antibiotics. To determine the minumum number of mutational paths or events required to rule out the possibility that an observed lack of transition bias might be due to small sample size, we downsampled our data sets as follows. For each of the Basel and Manson data sets separately, we randomly sampled *x* mutational paths or events from the data set without replacement and calculated the fraction of these paths or events that were transitions (we calculate this fraction, rather than the transition:transversion ratio, to avoid division by zero) and the associated 95% binomial confidence interval. We repeated this 10^4^ times for each value of *x* and calculated the average fraction of transitions, the minimum of the lower 95% confidence intervals, and the maximum of the upper 95% confidence intervals. For mutational paths, we varied *x* from 2 to the maximum number of mutational paths in each data set, and for mutational events, we varied *x* from 2 to 800. We then determined the minimum value of *x* for which the minimum of the 95% confidence intervals exceeded 1/3, which is the expected fraction of transitions under the null model (corresponding to a transition:transversion ratio of 0.5). This is the minimum number of mutational paths or events required to exclude the possibility that an observed lack of transition bias at a given value of *x* is due to small sample size, given the transition bias in the full data set. For the Basel data set, this minimum number of mutational paths was 44, and the minimum number of mutational events was 14. In the Manson data set, the minimum number of mutational paths was 118, and the minimum number of mutational events was 12. No antibiotic was associated with more than these minimum numbers of mutational paths in either the Basel or Manson data sets (S6A and S6B Fig). However, we had at least the minimum number of mutational events for all antibiotics and combinations except for amikacin + capreomycin and mutations conferring resistance only to capreomycin in the Basel data set (S6C and 6D Fig).

## Supporting information

S1 Data Summary data visualized in Figs 2 and 3 and S1–S6 Figs. (XLSX)

S2 Data One hundred ninety-six resistance-conferring mutations. For each mutation, this data set includes the mutation’s genomic position, the amino acid change it causes (if any), the antibiotic it confers resistance to, and supporting evidence that the mutation confers resistance, including references to pertinent literature.
(XLSX)

S3 Data 9,351 publicly available *MTB* genomes. For each genome, this data set includes the EMBL biosample ID and accession number. *MTB*, *Mycobacterium tuberculosis*; EMBL, European Molecular Biology Laboratory.
(XLSX)

S4 Data The Basel data set. For each of the 152 mutations, this data set includes the mutation’s genomic position, the amino acid change it causes (if any), the antibiotic it confers resistance to, the number of times it was found in the 9,351 *MTB* genomes, and the inferred number of times it independently evolved (i.e., mutational events). *MTB*, *Mycobacterium tuberculosis*.
(XLSX)

S1 Fig The number of mutational paths associated with a given number of mutational events that are (A, B) transitions or (C, D) transversions. Mutational paths associated with more than 100 events are indicated with text.
(TIFF)

S2 Fig Comparison of mutational events in the Basel data set with those observed genome wide. (A) Relative rates of the six nucleotide pair mutations, for mutational events in the Basel data set of antibiotic resistance mutations and genome-wide among 9,351 *MTB* strains. Transitions are indicated with bold text. Rates adjusted for GC content (Materials and methods). (B) Distribution of events per path in the Basel data set (open circles) and genome-wide (filled circles). *MTB*, *Mycobacterium tuberculosis*.
(TIFF)

S3 Fig The relative rates of each nucleotide pair mutation among events in the Basel data set are not statistically significantly different from those observed genome wide, given the distribution of events per path in the Basel data set. Bars show the distributions of the relative rates of each nucleotide pair mutation, observed across 10^5^ random resamplings of the genome-wide mutations, controlling for the number of events per path. In each resampling, we chose 152 mutational paths at random from the 305,316 mutational paths in the genome-wide data set and randomly assigned the number of events to each path according to the distribution of events per path from the Basel data set. Vertical dashed lines indicate the relative rates of each nucleotide pair mutation among events in the Basel data set (i.e., the height of the white bars in S2A Fig).
(TIFF)

S4 Fig Rarefaction analysis of the Basel and Manson data sets. Symbols represent the mean fraction of paths that are transitions given a random sample of events, drawn with replacement from the Basel and Manson data sets. Sampling was performed 10^5^ times for each number of sampled events (each position along the x-axis). Error bars indicate one standard deviation. We show the fraction of sampled paths that are transitions, rather than the transition:transversion ratio, to avoid division by zero when the number of sampled events is small. The upper horizontal dashed lines show the fraction of events that are transitions in the entire Basel and Manson data sets. The middle dashed lines show the fraction of paths that are transitions in the entire Basel and Manson data sets. The lower dashed lines show a fraction of 1/3, which corresponds to a transition:transversion ratio of 0.5.
(TIFF)

S5 Fig The average fraction of transitions (open circles) with the minimum and maximum of the lower and upper 95% binomial confidence intervals (dashed lines) relative to the number of sampled paths or events from the Basel or Manson data sets (x-axis). Open circles and dashed lines are derived from 10^4^ replications per number of sampled paths or events. The horizontal line indicates the null expectation of a fraction of transitions equal to 1/3 (i.e., transition:transversion ratio = 0.5), and the vertical line indicates the minimum number of mutational paths or events required for the minimum lower bound on the 95% confidence interval to exceed 1/3.
(TIFF)

S6 Fig The numbers of mutational paths and events that confer resistance to individual or multiple antibiotics in the Basel and Manson data sets. (TIFF)

S1 Table Transition:transversion ratios at the path and event level for rifampicin resistance. Data from fluctuation assays for rifampicin resistance at three drug concentrations in two *MTB* strains (CDC-1551 and HN878) show stronger transition bias among events than paths (data from S3 Table [37]). Similarly, in the Basel and Manson data sets, the event-level transition:transversion ratios for rifampicin resistance mutations were 1.64 and 2.36, whereas the path-level ratios were 0.54 and 0.60, respectively.
(DOCX)

## Abbreviations

MTB: *Mycobacterium tuberculosis*
