# Supplementary figures and images for: Transition bias influences the evolution of antibiotic resistance in Mycobacterium tuberculosis
Source: PLoS Biol. 2019 May 13;17(5):e3000265. doi: 10.1371/journal.pbio.3000265 (PMC6532934; doi:10.1371/journal.pbio.3000265)

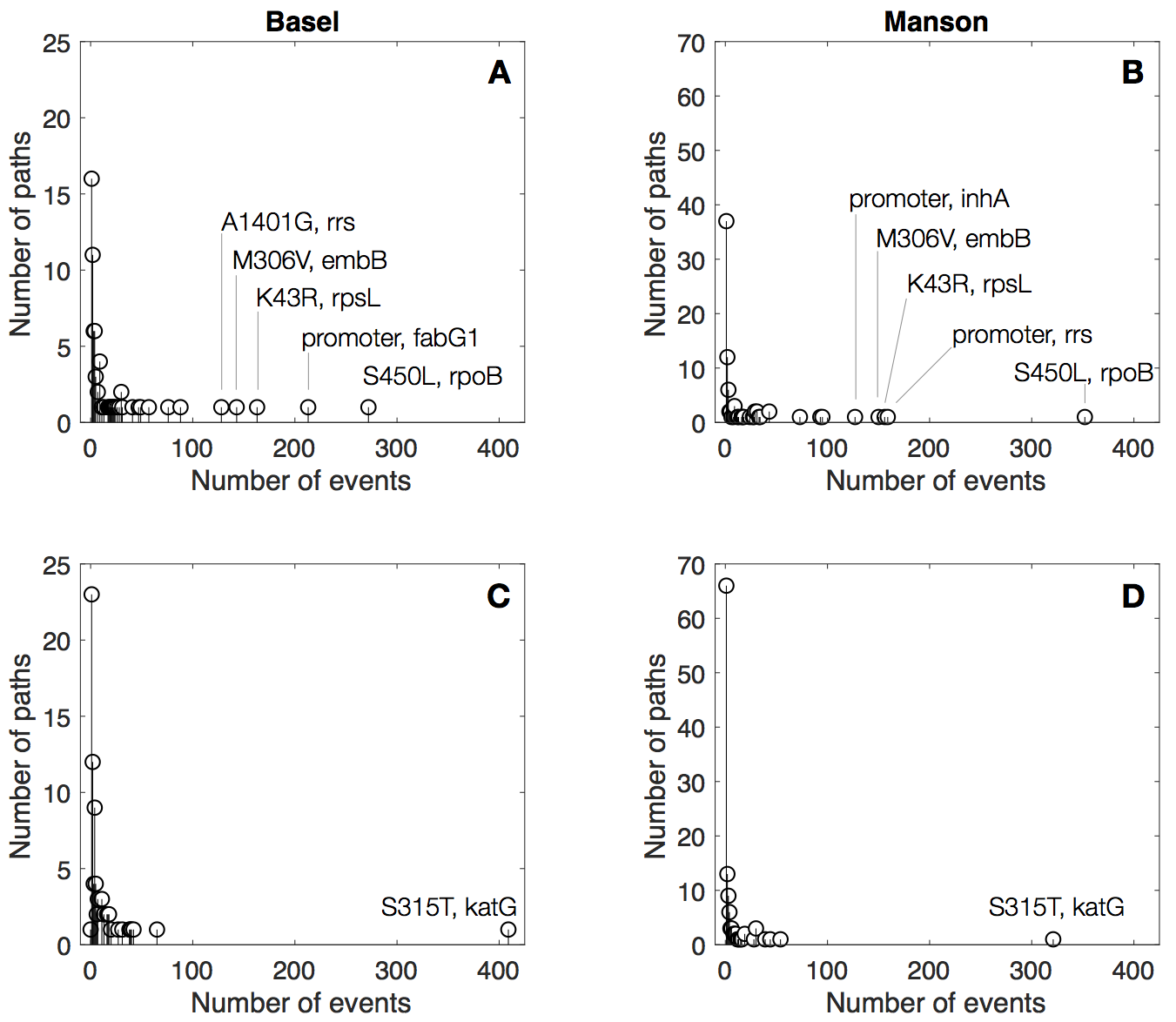

Supplement: S1 Fig — Mutational paths associated with more than 100 events are indicated with text. (TIFF) [file pbio.3000265.s005.tiff]

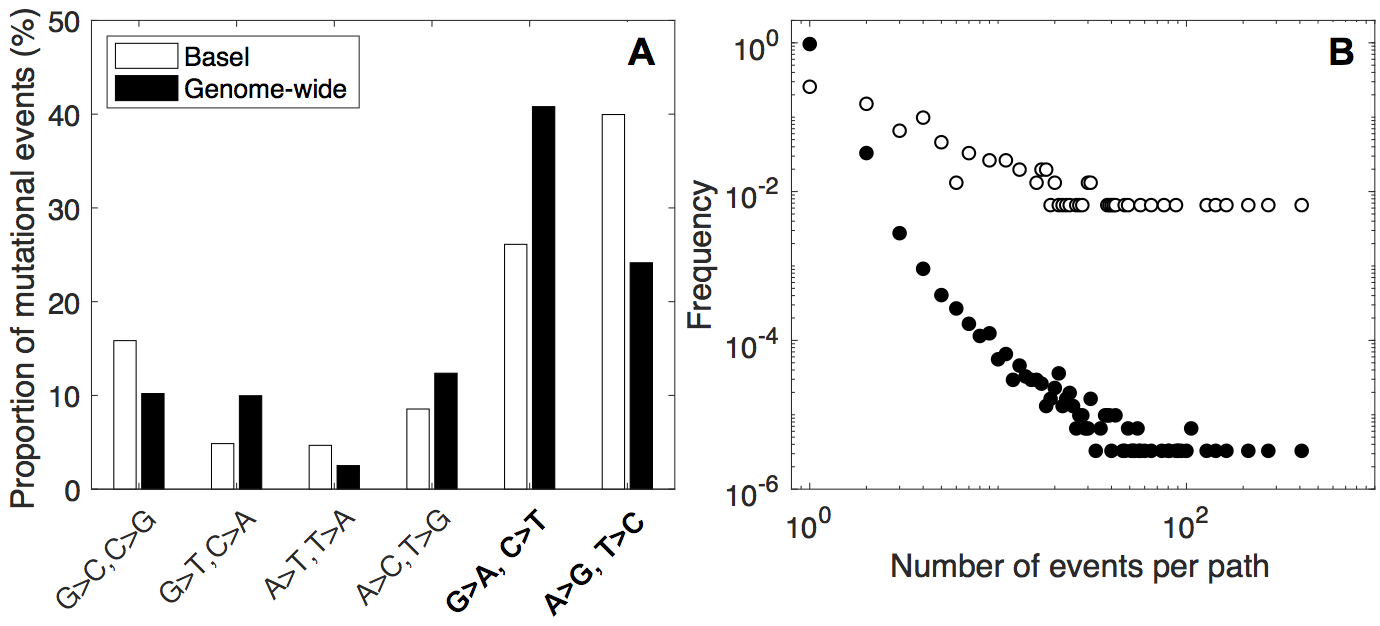

Supplement: S2 Fig — (A) Relative rates of the six nucleotide pair mutations, for mutational events in the Basel data set of antibiotic resistance mutations and genome-wide among 9,351 MTB strains. Transitions are indicated with bold text. Rates adjusted for GC content (Materials and methods). (B) Distribution of events per path in the Basel data set (open circles) and genome-wide (filled circles). MTB, Mycobacterium tuberculosis. (TIFF) [file pbio.3000265.s006.tiff]

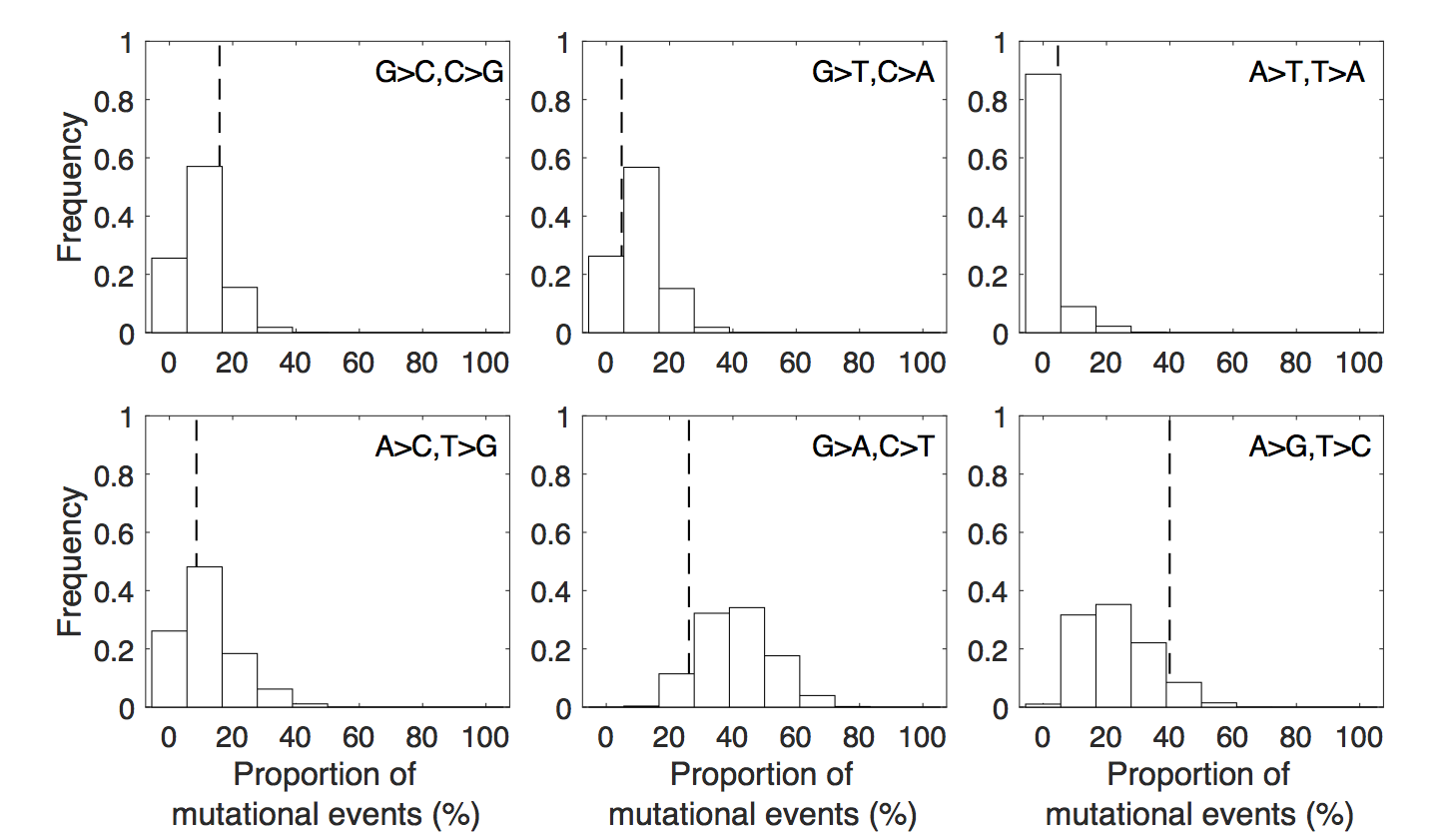

Supplement: S3 Fig — Bars show the distributions of the relative rates of each nucleotide pair mutation, observed across 105 random resamplings of the genome-wide mutations, controlling for the number of events per path. In each resampling, we chose 152 mutational paths at random from the 305,316 mutational paths in the genome-wide data set and randomly assigned the number of events to each path according to the distribution of events per path from the Basel data set. Vertical dashed lines indicate the relative rates of each nucleotide pair mutation among events in the Basel data set (i.e., the height of the white bars in S2A Fig). (TIFF) [file pbio.3000265.s007.tiff]

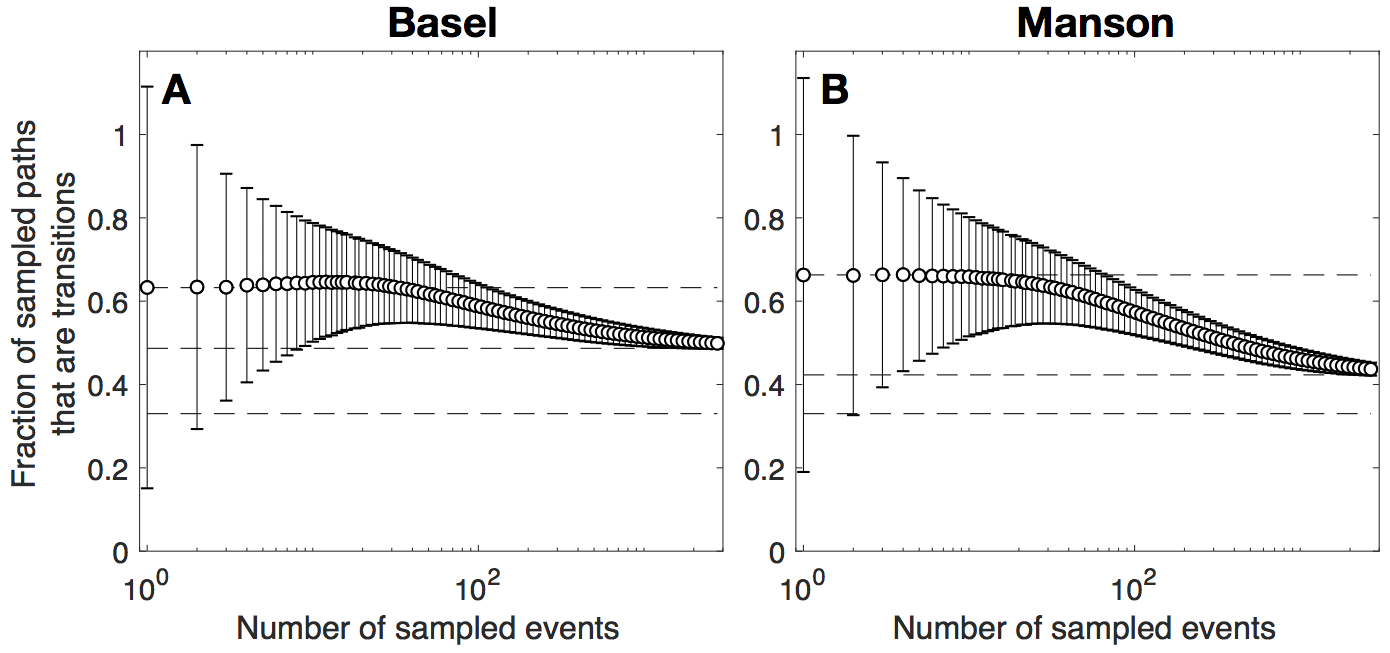

Supplement: S4 Fig — Symbols represent the mean fraction of paths that are transitions given a random sample of events, drawn with replacement from the Basel and Manson data sets. Sampling was performed 105 times for each number of sampled events (each position along the x-axis). Error bars indicate one standard deviation. We show the fraction of sampled paths that are transitions, rather than the transition:transversion ratio, to avoid division by zero when the number of sampled events is small. The upper horizontal dashed lines show the fraction of events that are transitions in the entire Basel and Manson data sets. The middle dashed lines show the fraction of paths that are transitions in the entire Basel and Manson data sets. The lower dashed lines show a fraction of 1/3, which corresponds to a transition:transversion ratio of 0.5. (TIFF) [file pbio.3000265.s008.tiff]

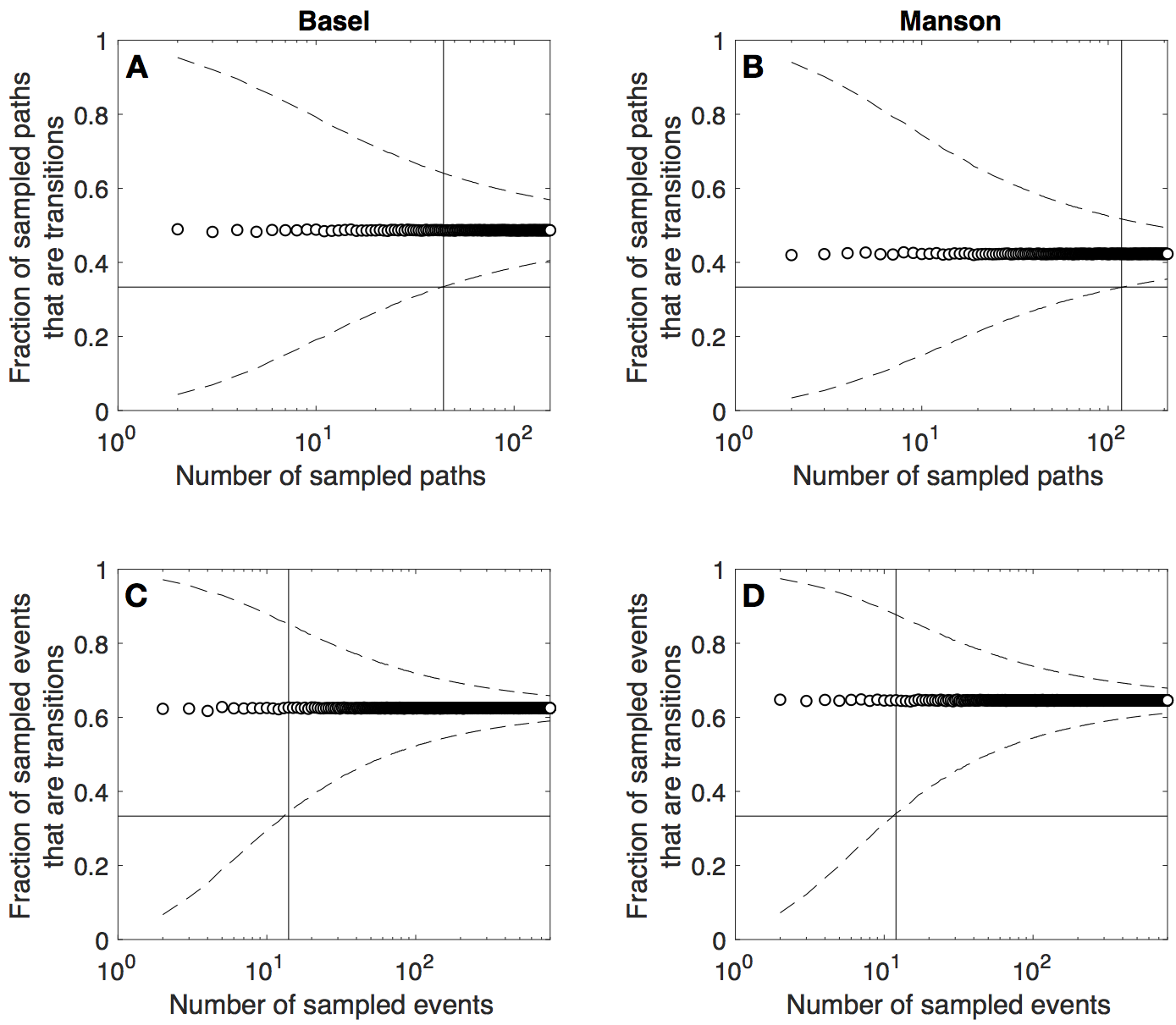

Supplement: S5 Fig — Open circles and dashed lines are derived from 104 replications per number of sampled paths or events. The horizontal line indicates the null expectation of a fraction of transitions equal to 1/3 (i.e., transition:transversion ratio = 0.5), and the vertical line indicates the minimum number of mutational paths or events required for the minimum lower bound on the 95% confidence interval to exceed 1/3. (TIFF) [file pbio.3000265.s009.tiff]

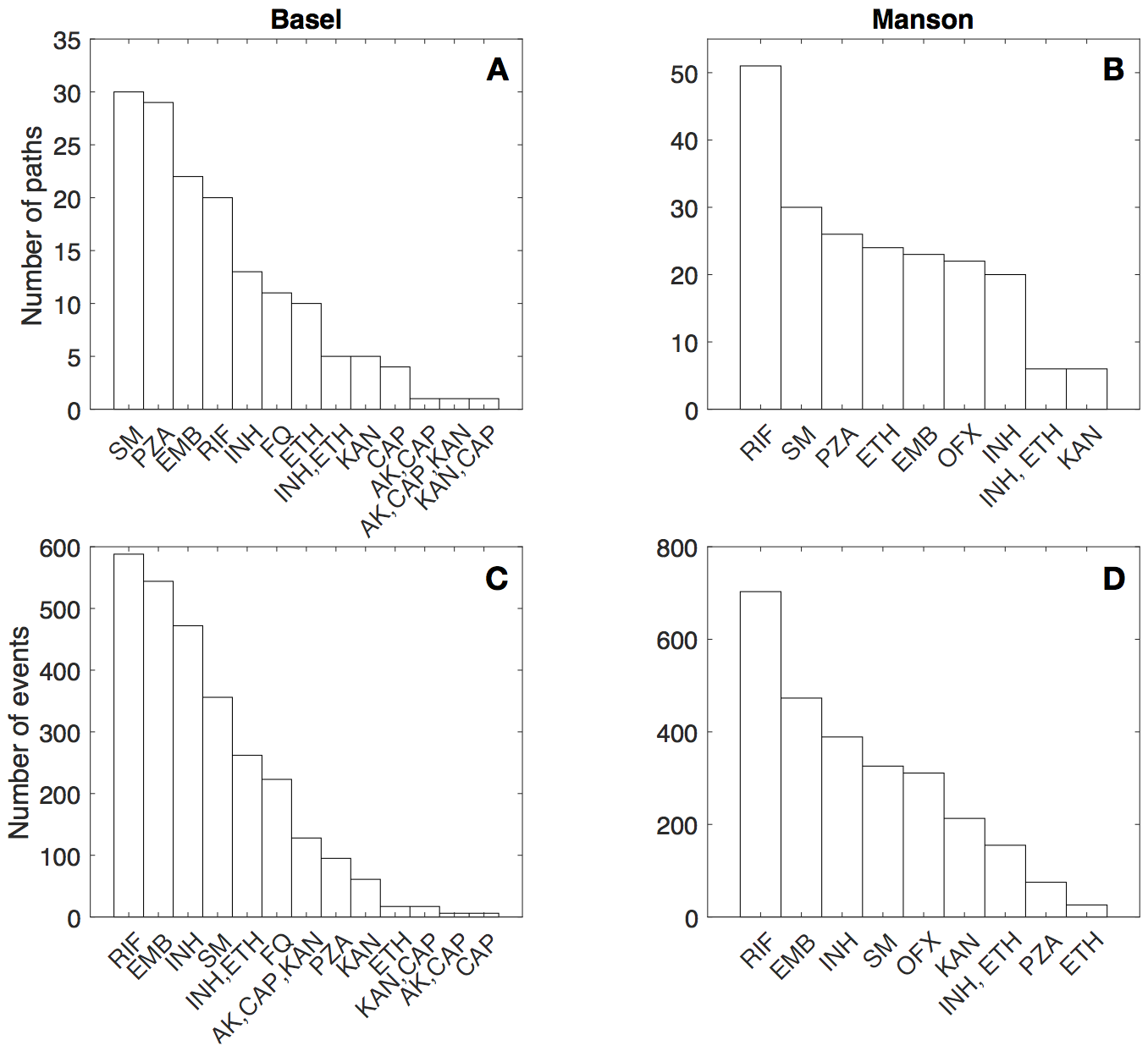

Supplement: S6 Fig — (TIFF) [file pbio.3000265.s010.tiff]
