# Supplementary material for: Transition bias influences the evolution of antibiotic resistance in Mycobacterium tuberculosis
Source: PLoS Biol. 2019 May 13;17(5):e3000265. doi: 10.1371/journal.pbio.3000265 (PMC6532934; doi:10.1371/journal.pbio.3000265)
Supplement: S1 Table — Data from fluctuation assays for rifampicin resistance at three drug concentrations in two MTB strains (CDC-1551 and HN878) show stronger transition bias among events than paths (data from S3 Table [37]). Similarly, in the Basel and Manson data sets, the event-level transition:transversion ratios for rifampicin resistance mutations were 1.64 and 2.36, whereas the path-level ratios were 0.54 and 0.60, respectively. (DOCX) [file pbio.3000265.s011.docx]

**Table S1 Transition:transversion ratios at the path and event level for rifampicin resistance. Data from fluctuation assays for rifampicin resistance at three drug concentrations in two MTB strains (CDC-1551 and HN878) shows stronger transition bias among events than paths (data from Supplementary Table 3 in Ford et al. [37]). Similarly, in the Basel and Manson datasets, the event-level transition:transversion ratios for rifampicin resistance mutations were 1.64 and 2.36, whereas the path level ratios were 0.54 and 0.60, respectively.**

| **Drug concentration (μg/mL)** | **CDC-1551** | | **HN878** | |
| --- | --- | --- | --- | --- |
|  | **Ti:Tv, paths** | **Ti:Tv, events** | **Ti:Tv, paths** | **Ti:Tv, events** |
| 0.5 | 4:6 = 0.67 | 35:23 = 1.52 | 4:9 = 0.44 | 36:24 = 1.50 |
| 2 | 4:4 = 1.00 | 34:17 = 2.00 | 4:5 = 0.80 | 54:12 = 4.50 |
| 5 | 3:4 = 0.75 | 30:15 = 2.00 | 4:3 = 1.33 | 48:15 = 3.20 |
